# Hydrologic Alteration and Enhanced Microbial Reductive Dissolution of Fe(III) (hydr)oxides Under Flow Conditions in Fe(III)-Rich Rocks: Contribution to Cave-Forming Processes

**DOI:** 10.3389/fmicb.2021.696534

**Published:** 2021-07-14

**Authors:** Kayla A. Calapa, Melissa K. Mulford, Tyler D. Rieman, John M. Senko, Augusto S. Auler, Ceth W. Parker, Hazel A. Barton

**Affiliations:** ^1^Department of Biology, University of Akron, Akron, OH, United States; ^2^Integrated Bioscience, University of Akron, Akron, OH, United States; ^3^Department of Geosciences, University of Akron, Akron, OH, United States; ^4^Instituto do Carste, Belo Horizonte, Brazil; ^5^Planetary Protection Center of Excellence, NASA Jet Propulsion Laboratory, Pasadena, CA, United States

**Keywords:** cave (speleogenic) and alluvial deposits (formations), iron reduction bacteria, hydrology and water, *Desulfosporosinus*, *Veillonella*

## Abstract

Previous work demonstrated that microbial Fe(III)-reduction contributes to void formation, and potentially cave formation within Fe(III)-rich rocks, such as banded iron formation (BIF), iron ore and canga (a surficial duricrust), based on field observations and static batch cultures. Microbiological Fe(III) reduction is often limited when biogenic Fe(II) passivates further Fe(III) reduction, although subsurface groundwater flow and the export of biogenic Fe(II) could alleviate this passivation process, and thus accelerate cave formation. Given that static batch cultures are unlikely to reflect the dynamics of groundwater flow conditions *in situ*, we carried out comparative batch and column experiments to extend our understanding of the mass transport of iron and other solutes under flow conditions, and its effect on community structure dynamics and Fe(III)-reduction. A solution with chemistry approximating cave-associated porewater was amended with 5.0 mM lactate as a carbon source and added to columns packed with canga and inoculated with an assemblage of microorganisms associated with the interior of cave walls. Under anaerobic conditions, microbial Fe(III) reduction was enhanced in flow-through column incubations, compared to static batch incubations. During incubation, the microbial community profile in both batch culture and columns shifted from a Proteobacterial dominance to the Firmicutes, including Clostridiaceae, Peptococcaceae, and Veillonellaceae, the latter of which has not previously been shown to reduce Fe(III). The bacterial Fe(III) reduction altered the advective properties of canga-packed columns and enhanced permeability. Our results demonstrate that removing inhibitory Fe(II) via mimicking hydrologic flow of groundwater increases reduction rates and overall Fe-oxide dissolution, which in turn alters the hydrology of the Fe(III)-rich rocks. Our results also suggest that reductive weathering of Fe(III)-rich rocks such as canga, BIF, and iron ores may be more substantial than previously understood.

## Introduction

The Southern Espinhaço Mountain Range (SE) of southeastern Brazil contains commercially important, high-grade iron ore hosted by the Serra da Serpentina Group, a stratigraphic unit which includes the iron-rich Serra do Sapo Formation (Auler et al., 2019). These sedimentary units were formed by the precipitation of Fe(III) and Si phases from solution during the Proterozoic Eon (Weber et al., 2006; Rosière et al., 2019; Silveira Braga et al., 2021). Iron ores can include magnetite (Fe_3_O_4_), hematite (α-Fe_2_O_3_), or a ferric oxyhydroxide like goethite (α-FeOOH) or limonite (FeO(OH)⋅n(H_2_O), and high-grade ore averages between 60 and 67% Fe (Dorr, 1964; Beukes et al., 2003; Rolim et al., 2016). The SE and Quadrilátero Ferrífero (Iron Quadrangle; QF) located ∼150 km south of SE, contain abundant Fe(III)-rich minerals, which can be found in intact banded iron formation (BIF), Si-depleted ore, and canga rock (Beukes et al., 2003; Souza et al., 2015; Auler et al., 2019). BIF contains alternating bands of quartz (SiO_2_) and either magnetite or hematite that range from a few millimeters to a few centimeters in thickness, and averages between 15 and 38% iron (Smith, 2015; Rolim et al., 2016). Canga is a brecciated duricrust containing clasts of iron oxide (usually BIF) with an iron-oxide cement matrix that averages between 57 and 62% iron (Dorr, 1964; Spier et al., 2007; Gagen et al., 2019). Canga contains the most poorly crystalline Fe(III) of the three major phases (i.e. canga, BIF, and ore), with goethite the most prominent mineral phase (Parker et al., 2013).

Canga and BIF are generally considered highly resistant to both mechanical and chemical weathering at pH ≥ 3 (Dorr, 1964; Johnson et al., 2012; Auler et al., 2014; Spier et al., 2018; Gagen et al., 2019), and canga covers the slopes and valleys of the SE region. Yet this area is also associated with hundreds of caves (iron formation caves; IFCs) that form mostly at the BIF/canga boundary (Auler et al., 2019). The identification of these caves suggests that processes leading to Fe(III) weathering and removal increase porosity at the canga/BIF interface, despite the resistance of both types of rocks to dissolution. At circumneutral pH, Fe solubility can be enhanced by microbially mediated reductive dissolution of Fe(III) phases to relatively soluble Fe(II). This activity may facilitate the mass transport necessary for the increased porosity and the formation of the observed IFCs (Parker et al., 2013, 2018). In support of this hypothesis, while the walls of the IFCs are lined with a hard, oxidized layer of canga, the interior (approximately 3 cm behind) of the wall surface contains a soft, gooey, water-saturated material that contains abundant microbial cells (Parker et al., 2018). Given the inhibition of Fe-reduction by oxygen, we wondered whether this material was involved in promoting Fe-reduction and increased porosity, leading to formation of the IFCs. Canga is a rather porous media, and active vertical percolation of water occurs during rainfall, the patterns of which can be irregular, depending on season (Mesquita et al., 2017; Parker et al., 2018). As such, intermittent periods of extensive water circulation around and within caves and their hosting rocks can occur, followed by water stagnation or dry periods.

Prior enrichment of canga-associated microorganisms from IFCs demonstrated that the microbial communities present were capable of Fe(III) reduction to extents that could contribute to IFC formation (Parker et al., 2018), but Fe(II) that accumulates during Fe(III) (hydr)oxide reduction can adsorb to Fe(III) phase surfaces and induce mineral (trans)formations (Roden et al., 2000; Benner et al., 2002; Hansel et al., 2003, 2005; Gonzalez-Gil et al., 2005). These consequences of Fe(III) reduction could self-limit further Fe(III) (hydr)oxide reduction, although subsurface water flow could help overcome these limitations by advective transport of Fe(II) (Gonzalez-Gil et al., 2005; Minyard and Burgos, 2007; Wefer-Roehl and Kübeck, 2014). Additionally, it remained unclear if the extents of microbiological Fe(III) (hydr)oxide dissolution observed were sufficient to induce hydrologic alterations that would culminate in cave formation. To understand whether this hydrologic flow could influence Fe-reduction rates and enhance IFC formation we compared batch cultures [where Fe(II) will accumulate] to columns [where Fe(II) is removed via flow] to evaluate how water flow influenced microbiologically mediated Fe(III) reduction, and whether such activity could influence the hydraulic properties of canga.

## Materials and Methods

### Sample Collection and Preparation for Batch Incubations and Column Experiments

Five IFCs were sampled in the SE region of Brazil in December 2018. The cave designations are: CSS-0009, CSS-0080, CSS-0010, CSS-0107, and CSS-0074. Authorization for sampling during the destruction of these caves through mining had been approved by the Brazilian environmental agency, and the sampling was part of a final recovery effort prior to mining. Canga was collected from the interior of a cave that was forming at a BIF-canga interface. Large (0.5–5.5 kg) chunks of canga were removed from cave walls using an electric demolition hammer during the final sampling effort prior to cave destruction and placed in plastic bags. The soft, microbial-rich material behind the walls (*sub muros*) was collected by first removing the rigid oxide layer and then using a sterile garden shovel to place this material in sterile glass jars and sealed with plastic tape to maintain anaerobic/microaerobic conditions. Samples were placed in a refrigerator upon return from the field. Collected canga was pulverized for column experiments by cutting large chunks into smaller pieces with a water-cooled trim saw with a 25 cm blade, which were then processed through a ball mill (SPEX Industries, Inc., Metuchen, NJ, United States) until all the material passed through a 1.44 mm sieve. This pulverized canga was sterilized by autoclaving at 121°C for 15 min, allowing a 1 h recovery, and then autoclaved again to assure deactivation of spore-forming bacteria. This sterilized canga was then dried at 65°C for 14–16 h in an oven.

### Batch Incubations

Preparation of batch incubations was carried out in a Coy anaerobic glove bag (Coy Laboratory Products, Inc., Grass Lake, MI, United States) filled with 3-5% H_2_, balance N_2_. Synthetic porewater (SPW) composition was based on characterization of IFC porewaters, and contained 5 mM CaCl_2_, 0.1 mM Na_2_SO_4_, and 0.1 mM KH_2_PO_4_. Sodium lactate (5.0 mM) was included as an electron donor and carbon source, and the pH of the SPW was adjusted to either pH 4.75 or pH 6.8 with HCl or NaOH, respectively, which were chosen to approximate the pH of porewaters we have observed in IFCs (Parker et al., 2018). O_2_ was removed from the SPW by bringing the liquid to nearly boiling, then cooling under a stream of N_2_ gas for 45 min. Once cooled, SPW-containing bottles were sealed, transferred to the anaerobic glove bag, and filter-sterilized with a 0.2 μm PES filter (VWR, Radnor, PA, United States) until use in incubation bottles or column experiments. Batch sediment incubations contained 20 g of pulverized canga (equivalent to approximately 120 mmol Fe(III)) and 60 mL of SPW in 160 mL serum bottles that were sealed with butyl rubber stoppers held in place with aluminum crimp seals. Where appropriate, 5 g of *sub muros* material and associated microbial community was used as inoculum for non-sterile incubations. Incubations were carried out in triplicate.

### Column Assembly and Operation

All materials used for packing columns were acid-washed and sterilized prior to use, and all columns (10 cm × 1 cm Econo-Columns; Bio-Rad Laboratories, Hercules, CA, United States) were packed and operated in a Coy anaerobic glove bag as described. Approximately 2 g of 3 and 2 mm diameter glass beads were placed at the bottom of the column, respectively, to prevent sediment clogging. The uninoculated columns were packed with pulverized sterile canga using a “lift” technique: a 2 g lift of canga was added, followed by the injection of 200–500 μL SPW (described above) through bottom of the column. After the addition of each lift, the columns were tapped to remove air bubbles, and sediments were allowed to settle for 1–2 min. A suspension of *sub muros* was prepared with 8 g pulverized canga and 8 g *sub muros* in 6 mL SPW, which was added 250 μL at a time after each lift of canga/SPW during column packing. The final amount of *sub muros* added was ∼2 mL, which resulted in ∼9.2 × 10^7^ cells/g column material (as determined by direct cell counting; see below). All columns received 4 g of 2 mm beads followed by 2 g of 3 mm beads added to on top of the sediments to prevent clogging. After packing, columns contained a total of 16 g of solids and 7 mL liquid (either SPW only in uninoculated columns or SPW and microbial suspension in inoculated columns). All column incubations were carried out in triplicate in the anaerobic glove bag for 14 days prior to the first sampling.

Synthetic porewater was delivered to columns in an upward flow during sampling using a Masterflex L/S Precision Variable-Speed Console peristaltic pump with Masterflex L/S 8-Channel Pump Head (Cole-Parmer, Vernon Hills, IL, United States), and 1.6 mm inner diameter Tygon tubing (Fischer Scientific, Pittsburgh, PA, United States). The columns were subsequently incubated statically for 7 days at room temperature between sampling events, whereupon 3–5 column void volumes of SPW were passed through at a flow rate of 0.2 mL/min, and during this period samples were collected from each column volume for measurement of pH, sulfate concentration, and dissolved Fe(II) concentration (described below). At the completion of the experiments, breakthrough curves were determined with 1 mM NaBr-amended (Acros Organics, Morris, NJ, United States) SPW at a flow rate of 0.2 mL/min, and samples were periodically collected for bromide quantification (described below). At the conclusion of the experiments, columns were deconstructed and sediments were removed for analysis of the microbial communities and quantification of total Fe(II) and microbial cells.

### Sample Processing and Analytical Techniques

Column effluent samples for dissolved Fe(II) quantification were preserved in 0.5 M HCl, while those intended for measurement of pH, sulfate, and bromide were untreated. Solids were removed from the samples by centrifugation at 12,100 × *g* for 5 min. To measure solid-associated Fe(II) in column sediments, approximately 0.5 g of material was placed in microcentrifuge tubes with 1 mL 0.5 M HCl (Lovley and Phillips, 1987) and solids were then separated from the solution by centrifugation before measurement of Fe(II) in the supernatant. Fe(II) was quantified by ferrozine assay (Stookey, 1970). pH was measured using a SevenGo Pro pH/Ion meter (Mettler Toledo, Columbus, OH, United States). Sulfate, bromide, phosphate, nitrate, and chloride were measured by ion chromatography with a Dionex DX-120 System with an IonPac AS22 Column and conductivity detection (Thermo Fisher Scientific, Waltham, MA, United States). To analyze the mineralogy of the column at the end of the experiment, approximately 1 mL of column contents were dried in a closed container with CaCl_2_ (Acros Organics, Morris, NJ, United States) as a drying agent under anaerobic conditions. Mineral characterization of the homogenized rock was determined by X-Ray Diffraction (XRD) with a Rigaku Ultima IV (Rigaku, Woodlands, TX, United States). Samples were analyzed with a 2θ between 5° and 70° and diffraction patterns were compared to those standards available on the American Mineralogist Crystal Structure Database (Downs and Hall-Wallace, 2003).

### Microbial Community Analysis

Microbial cells in the *sub muros* inoculum, batch incubations, and in the columns at the conclusion of the experiments were enumerated by direct cell counting. Briefly, ∼1 g samples were collected while the columns remained in the anaerobic glove bag and mixed with 1 mL of filter-sterilized Dulbecco pH 7.4 phosphate buffered saline (PBS) (Thermo Fisher Scientific, Waltham, MA, United States) and vigorously shaken by hand to mix the contents. One hundred microliter of this mixture was then added to 9.9 mL sterile PBS and 1X thiazole green DNA stain (Biotium, Fremont, CA, United States). This mixture was incubated in the dark for 30 min at room temperature and then filtered onto a 0.2 μm GTBP Isopore polycarbonate membrane filter via a Millipore stainless steel filtration unit (Millipore Sigma, Darmstadt, Germany). The filter was removed and placed on a glass slide with 25 μL SlowFade Gold Antifade Mountant with DAPI (Thermo Fisher Scientific, Waltham, MA, United States) and cells were counted using a BX53 fluorescent microscope (Olympus America Inc., Center Valley, PA, United States). The total cell number was calculated based on 100 fields-of-view (FOV) at 1,000× magnification, with the average number of cells per FOV multiplied by the total dilution factor, area of the filter membrane, and standardized against the absolute weight of the material/g (Hershey et al., 2018).

For DNA analyses, genomic DNA was extracted from samples using either then DNeasy PowerLyzer PowerSoil or PowerBiofilm Kit (Qiagen, Germantown, MD, United States). Subsamples from the triplicate batch incubations were pooled before DNA extraction. PCR amplification of the 16S rRNA V3 and V4 regions using the primers 806R (5′-GGA CTA CHV GGG TWT CTA AT-3′) and 515F (5′-GTG CCA GCM GCC GCG GTA A-3′), with samples identified using unique barcodes along with Illumina adapter sequences (Integrated DNA Technologies, Coralville, IA, United States). Amplification was using a Mastercycler Nexus Gradient (Eppendorf, Enfield, CT, United States), including a 3 min 94°C hot start, followed by 30 cycles of: denaturing at 94°C for 45 s, annealing at 50°C for 60 s, and then a 72°C extension for 90 s, followed by a final extension step at 72°C for 10 min. The PCR products were gel purified, and quantified using a Qubit dsDNA HS Assay Kit (Life Technologies, Waltham, MA, United States). Samples were then sequenced on an Illumina MiSeq and de-multiplexed in QIIME2 (version 2020.2; Bolyen et al., 2019) using *cutadapt demux-*paired and a quality check was carried out using *q2-deblur-denoise-16S* and *quality-filter-q-score*. OTU picking and taxonomic assignments were completed using the *feature-classifier-classify-sklearn* (Bolyen et al., 2019).

## Results and Discussion

### Fe(III) Reduction in Static Incubations

**FIGURE 1:**
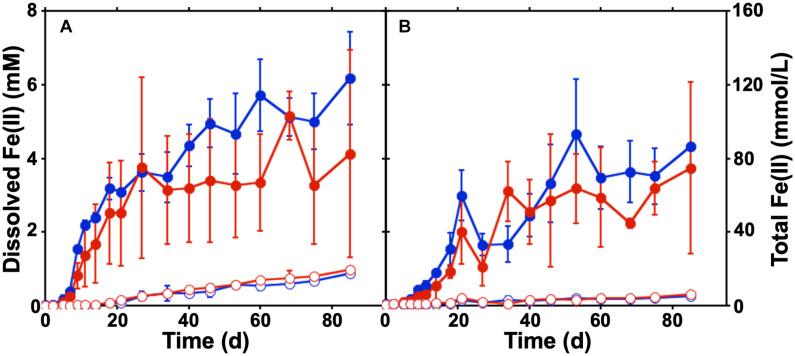
Batch cultures of Fe(III) reduction in SPW canga. The concentration of dissolved Fe(II) **(A)** and total Fe(II) **(B)** were measured under static conditions over 3 months. Comparisons were made between sterile canga (open circles) or canga inoculated with *sub muros* material (closed circles), with a basal SPW medium pH of 4.75 (red) or pH 6.8 (blue). Error bars represent the standard deviation of triplicate incubations.

To evaluate the canga-Fe(III) reducing activities of the microbial communities in the *sub muric* material, static batch incubations were conducted. Canga was provided as the Fe(III) source with SPW at a pH of 4.75 or 6.8, which matched the measured pH values *in situ* (Parker et al., 2018). Minimal Fe(II) was generated in uninoculated incubations, but accumulated in the *sub muros*-inoculated incubations at both pH 4.75 and 6.8 (Figure 1A). The concentration of dissolved Fe(II) that accumulated in the *sub muros-*inoculated batch incubations (approximately 5 mM) exceeded previous batch incubation work in which *Shewanella oneidensis* MR-1 was used to catalyze canga-Fe(III) reduction (less than 0.6 mM; Parker et al., 2013). Indeed, mean total Fe(II) concentration of *sub muros*-inoculated incubations exceeded 80 mmol/L (Figure 1B). In previous work, a maximum of 3% of canga-Fe(III) could be reduced by *S. oneidensis* MR-1 (Parker et al., 2013); however, greater extents of Fe(III) reduction have been observed by fermentative enrichments and isolates from canga by ourselves and other researchers (Parker et al., 2018; Gagen et al., 2019). The drivers of this enchanced reduction remain unclear at this time and represent a good target for future research.

**FIGURE 2:**
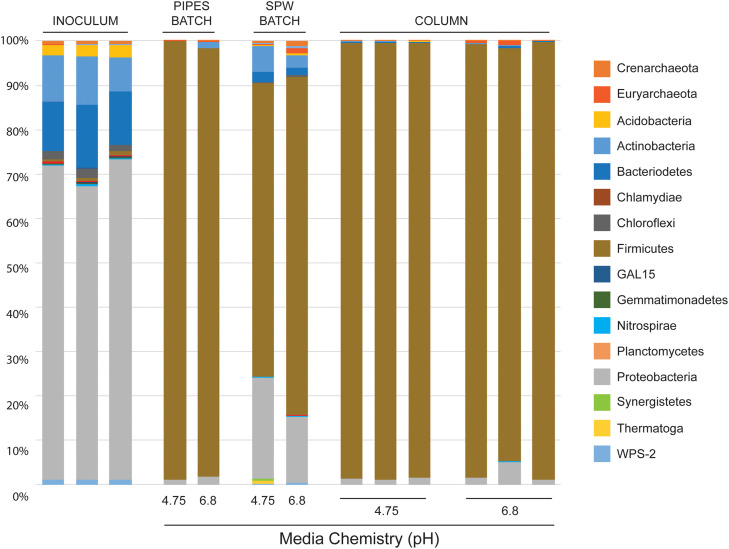
Illumina sequencing results of phylum-level community diversity in batch and column cultures. Illumina sequencing of *sub muros* inoculated samples at day 0 are shown (inoculum). The diversity in our previous batch culture experiments, where the basal media was buffered with PIPES is shown (indicated as PIPES BATCH; Parker et al., 2018), followed by the cultures presented here using SPW with lactate (SPW BATCH). Illumina data is also provided for each of the individual columns in the flow-through experiments (COLUMN). The basal pH of each media formulation at day 0 is shown (Media Chemistry pH).

In previous cultures using various Fe(III) mineral phases (including canga; Parker et al., 2018) we used a PIPES-buffered (also at pH 4.75 and 6.8) mineral salts medium for growth. In these cultures we saw a shift in microbial community structure from the Proteobacteria-dominated *sub muros* to one dominated by the Firmicutes, representing >97% of sequences (Figure 2). In these previous experiments, we had assumed that the shift in community structure had been driven in part by the high amount of organic carbon, while the closed nature of the experiment allowed H_2_ to accumulate and drive fermentative Fe-reduction by members of the Clostridia (Shah et al., 2014; Parker et al., 2018). We tested this hypothesis in this study, using a basal medium (SPW) and 5 mM lactate as a carbon source. Analysis of partial 16S rRNA gene sequences in the batch incubations after 85 days revealed a similar dominance by fermentative Firmicutes (Figure 2). Nonetheless, using SPW/lactate, the Proteobacteria remained abundant, comprising 23 and 15% of the sequences recovered from pH 4.75 and 6.8 incubations, respectively. We also saw a small, but significant population of Actinobacteria (6% at pH 4.75 and 3% at pH 6.8) and Bacteriodetes (2.5% only at pH 6.8) that had not been observed previously (Figure 2). The pH of the uninoculated controls averaged 5.44, regardless of whether the pH 4.75 or 6.8 SPW was used to initiate the experiment, suggesting that canga buffered the pH; however, in the inoculated batch cultures, the SPW/lactate pH 4.75 culture increased to pH 6.10, while the SPW pH 6.8 culture remained reasonably constant at pH 6.67. There was no dramatic change in pH of the cultures following the addition of *sub muros* at day 0. This suggests Fe(III) reduction through microbial activity likely raises the pH (Eq 1):

(1) $$\mathrm{Fe}{\left(\text{OH}\right)}_{3}+3{\text{H}}^{+}+{\text{e}}^{-}\to {\text{Fe}}^{2+}+3{\text{H}}_{2}\text{O}$$

The similarity in pH of the final culture conditions may explain the similarity of the final observed community profiles (Figure 2).

**FIGURE 3:**
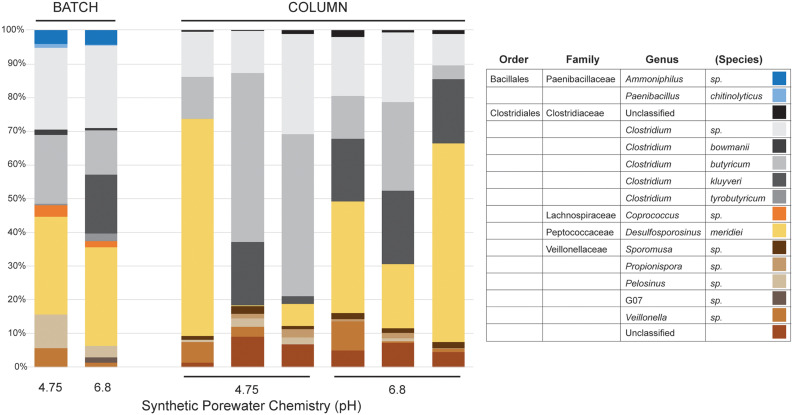
Illumina sequencing results of genus-level community diversity within the Firmicutes from the batch and column cultures. Only the SPW/lactate results are shown. The distribution of genera in the batch cultures (BATCH) and individual columns (COLUMN) are shown, along with the basal pH of the SPW at day 0 is shown. Given the myriad of Family- and Genera-level distributions within the Firmicutes, the Order/Family/Genus classification is provided for each identified species.

At the genus level within the dominant Firmicutes (Figure 3), the SPW/lactate batch cultures displayed a different structural diversity to our previous work. Previously, at pH 4.75 the batch cultures were dominated by members of the genus *Clostridium* (Family Clostridiales; 71%), with a small but significant representation by the *Desulfosporosinus* (Family Peptococcaceae; 6%), while at pH 6.8, the PIPES batch cultures were dominated by both the *Desulfosporosinus* (38%) and *Clostridium* (36%) (Parker et al., 2018). Both cultures also contained minor populations of the *Paenibacilli* (Family Bacillales; 3% at both pH 4.75 and 6.8). In the batch cultures presented here, we saw a similar dominance by members of the Clostridia (31% at pH 4.75 and 47% at pH 6.8), and *Desulfosporosinus* (20 and 24% at pH 4.75 and 6.8, respectively). The *Desulfosporosinus* sp. are normally associated with sulfate reduction, but have also been shown to reduce Fe(III) enzymatically (Senko et al., 2009; Sato et al., 2019). If not enzymatic, the production of a minor amount of sulfide could be sufficient to enable Fe(III) reduction via S as an electron shuttle (Hansel et al., 2015). Members of the *Paenibacilli*, which have recently been demonstrated to play an important role in iron oxide weathering in soils (including in Brazil; Loyaux-Lawniczak et al., 2019) were also represented at both pH 4.75 and 6.8 (4% of total diversity; Figure 3). Interestingly, we saw a higher percentage of members of the *Coprococcus* (Family Lachnospiraceae*;* 2%) at both pH 4.75 and 6.8. The genus *Coprococcus* includes strict anaerobes that play an important role in carbohydrate fermentation in the mammalian rumen, including lactate (Rainey, 2009). It is unclear as to why members of this genus would be enriched under the batch culture conditions; however, their growth is stimulated by fermentable carbohydrates, suggesting that the use of lactate may have enhanced their growth (Cotta and Forster, 2006; Rainey, 2009). Members of this genus have not been associated with Fe-reduction, or isolated from iron-rich environments, although the production of H_2_ during fermentation may contribute to the overall culture Fe-reduction conditions (Cotta and Forster, 2006; Parker et al., 2018). We also observed a significant representation by members of the Family Veillonellaceae, with 11% at pH 4.75 and 5% at pH 6.8 (Figure 3). Recently, genera within the Veillonellaceae, such as *Sporomusa* spp. and *Propionispora* spp., have been shown to carry out Fe(III) reduction (Sass et al., 2004; Kato et al., 2015); however, rather using respiratory Fe(III) reduction, the *Sporomusa* appear to use Fe(III) as an electron sink in acetogenesis (Igarashi and Kato, 2021).

### Fe(III) Reduction in Column Incubations

The underlying hypothesis of our work is that microbiological Fe(III) reducing activities are sufficient to induce porosity generation within the host rocks (i.e., canga, BIF, and iron ore); however, we have not observed hydrologic alterations of cave hosting rocks. Biogenic Fe(II) can limit the extent of Fe(III) (hydr)oxide reduction (Roden and Zachara, 1996; Urrutia et al., 1999; Roden and Urrutia, 2002; Roden, 2004, 2006), and induce mineralogical changes that would otherwise limit further Fe(III) reduction or limit the export of soluble Fe(II) (i.e., the formation of secondary minerals; Benner et al., 2002; Hansel et al., 2003, 2005). Nonetheless, the advective removal of biogenic Fe(II) as water flows through Fe(III) (hydr)oxide-rich rocks could enhance their reduction (Roden and Urrutia, 1999; Roden et al., 2000; Royer et al., 2004; Minyard and Burgos, 2007). For example, 95% of Fe(III) coating on sand was reduced over six months by *Shewanella putrefaciens* CN32 in flow-through columns, compared to 13% of the Fe(III) in batch incubations (Roden et al., 2000).

The climate regime in the SE area is highly seasonal, with over 80% of the ∼1,400 mm/year rainfall concentrated in November-March. Canga is a highly porous rock, with values between 24 and 29% (Costa and Sá, 2018), while the friable ore underneath the canga is highly impermeable with values as low as 10^–8^ m/s (Mesquita et al., 2017). Thus, rainfall infiltrates quickly through the canga towards the caves and then drains rapidly toward the surface, with very little retention of water, except in a few shallow internal ponds. Despite the robust Fe(III) reducing activity observed in the batch incubations (Figure 1; Parker et al., 2018), they do not mimic the hydrologic flow associated with the rocks of the SE or QF in which cave formation occurs with Fe(II) accumulating in the cultures. To mimic flow conditions in a laboratory setting, we packed canga into columns under conditions analogous to the batch incubations, and introduced flow into the system. This approach allowed us to answer the two major questions of this work: (1) does advective removal of biogenic Fe(II) enhance further canga-Fe(III) reduction and (2) are the Fe(III) reducing microbial activities associated with the *sub muric* material sufficient to induce hydrologic alterations to the host rock.

**FIGURE 4:**
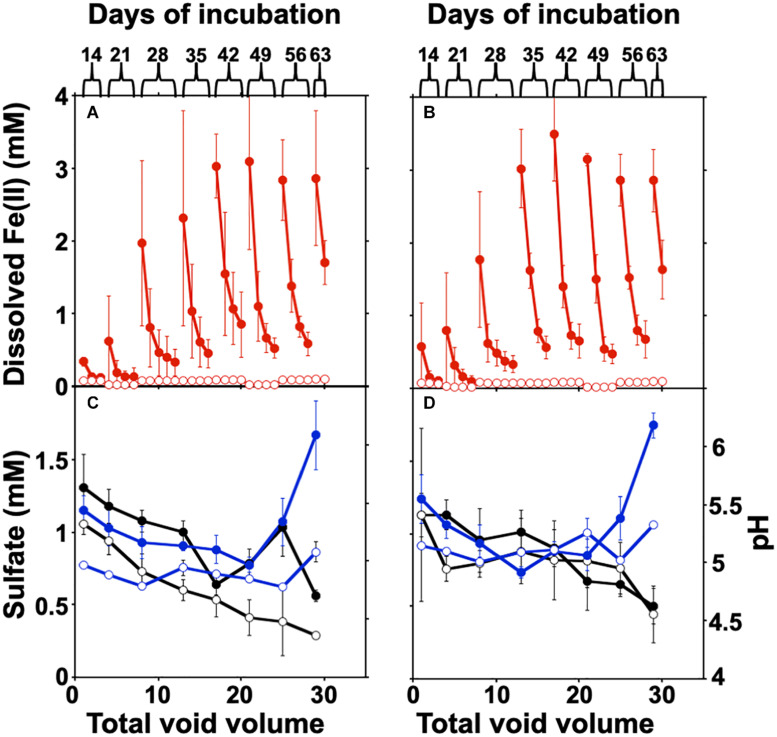
Fe(III) reduction and changes in sulfate and pH in the column experiments. **(A,B)** Columns were operated semi-continuously, and sulfate and pH were measured in each pore volume (four volumes) recovered after each static incubation. The column was disassembled after two column volumes at day 63 for post mortem analysis. Error bars represent one standard deviation of triplicate columns. The concentration of dissolved Fe(II) is shown at pH 4.75 **(A)** and 6.8 **(B)**. Sulfate concentrations (black) and pH (blue) in column effluents are shown in panels **(C)** (pH 4.75) and **(D)** (pH 6.8). The values for uninoculated columns are shown with open circles, with the *sub muros*-inoculated columns represented by closed circles.

The columns were packed with crushed canga alone, or with crushed canga mixed with *sub muric* material. The columns were incubated statically for 14 days, allowing Fe(III) reduction to initiate, before four column volumes of SPW were then passed through the column and collected separately for analysis of effluent chemistry (Figure 4). This process of static incubation followed by introduced flow was then repeated at 7 day intervals. Minimal dissolved Fe(II) was detected in the effluent of uninoculated control columns throughout the incubation (Figure 4), and effluent pH was ∼4.5–5.0, regardless of influent SPW pH. This is slightly lower than the values obtained in the batch experiments shown in Figure 1. In the inoculated columns, progressively higher concentrations of dissolved Fe(II) accumulated over the course of the incubation, with maximum Fe(II) concentrations of approximately 3 mM Fe(II) detected after the fifth round of static incubation at both pH 4.75 and 6.8 (Figures 4A,B).

The concentration of total Fe(II) (dissolved and solid-associated) produced in the columns incubated at either pH 4.75 or 6.8 SPW are shown in Table 1. A two-sample *t*-test assuming unequal variances suggested that there was no significant difference (*P* = 0.97) in total dissolved Fe(II) accumulation in the columns receiving SPW with pH 4.75 and 6.8 (Figure 4). The pH of the column effluent suggests that there was an increase in pH above 6.0 (Figure 4C), similar to the batch cultures. The increase in Fe(III) reduction in the pH 4.75 column as the experiment progressed may reflect a change in column community structure as the cultures move toward similar pH conditions. The increase in pH conditions is correlated with the increasing observation of *Clostridium kluyveri* (Figure 3) and may suggest either the selection of this species und these pH conditions, or a role in driving Fe(III)-reduction.

**TABLE 1:** Post mortem analysis of column contents.

	pH 4.75 with *sub muros*	pH 4.75 uninoculated	pH 6.8 with *sub muros*	pH 6.8 uninoculated
Total Fe(II) (μmol/g)	60 ± 15	4.4 ± 0.2	89 ± 19	4.3 ± 0.1
Cell abundances t = 0 (cell/g wet)	9.3 × 10^7^ ± 2.3 × 10^5^	N/D	9.1 × 10^7^ ± 1.8 × 10^5^	N/D
Cell abundances t = 63 (cell/g wet)	4.0 × 10^8^ ± 8.1 × 10^7^	N/D	4.2 × 10^8^ ± 4.7 × 10^7^	N/D
Fe(OH)_3_ removed as Fe^2+^ (mg)	38 ± 18	2.3 ± 0.2	40 ± 3.5	2.3 ± 0.01

While canga is composed mostly of goethite and poorly crystalline Fe(III) (hydr)oxides (Parker et al., 2013), if we assume dissolved Fe(II) is derived from Fe(OH)_3_, approximately 40 mg of Fe(OH)_3_ were reductively dissolved and exported as Fe(II) from the packing material of inoculated columns, with minimal export of Fe from uninoculated columns (Table 1). In the batch incubations, only ∼30 mg of Fe(OH)_3_ were reductively dissolved (Figure 1). After two porewater replacement events (21 days), the dissolved Fe(II) concentration in effluent from *sub muros*-inoculated columns exceeded 6 mM in total in pH 6.8 columns and over 5 mM total in pH 4.75 columns; iron reduction levels which only accumulated after 60 days continuous culture in batch incubations (Figures 1A, 4A,B). These results indicate that water flow enhances the reductive solubilization of Fe from canga and separation of the Fe(II) products from solid phases.

### Microbial Communities in Column Incubations

The microbial community composition in the batch incubations suggested that non-respiratory Fe(III) reduction could play a role in the observed iron reduction (Figure 3). To determine the extent of growth during column operation, we counted cells associated with the *sub muros* inoculum and at the conclusion of the column experiments. All the columns seeded with *sub muros* were initially inoculated at ∼9.2 × 10^7^ cells/g. At 63 days, the population had increased in the columns at pH 4.75 by 4.3×, with the cell number in the pH 6.8 column increasing 4.6×. These data suggested an increase in microbial growth, and indeed the higher cell number is the pH 6.8 columns matches a higher-level of Fe(III) reduction. No microbial cells were detected in the uninoculated controls (Table 1).

DNA extraction from the inoculated columns produced sufficient DNA for Illumina sequencing, but repeated attempts to extract DNA from the uninoculated columns failed, matching the observations by direct cell counting. Illumina sequencing of the microbial communities in the columns matched our observations in batch culture (Figures 2, 3); there had been a shift from dominance by the Proteobacteria, to dominance by members of the Firmicutes. At the genus level, the columns were similarly dominated by members of *Clostridium, Desulfosporosinus*, and *Veillonella*, which represented ≥90% of the identified partial 16S rRNA gene sequences (Figure 3); however, members of the *Paenibacilli* were not observed. There was some inter-column variability under each of the pH conditions, particularly in regard to the dominance of *Clostridium* relative to *Desulfosporosinus* (Figure 3). In the *Desulfosporosinus-*dominated columns, we saw a darkening of the column material, which could indicate sulfidogenesis, but there was no decrease in the effluent sulfate concentration over the course of the incubations (Figures 4C,D). This suggests that while members of the *Desulfosporosinus* are accumulating in these columns, they may be functioning as Fe(III) reducers. Indeed, members of this genus have been shown to be the primary Fe(III) reducers under oligotrophic conditions (Nixon et al., 2017; Bomberg et al., 2019). Fe(III) reduction is widespread among the Clostridia, including a strain of *Clostridium beijerinckii* (Dobbin et al., 1999; Lehours et al., 2010; Shah et al., 2014; List et al., 2019). Indeed, in our previous batch cultures were capable of extensive (in some cases, complete) Fe(III) reduction (Parker et al., 2018), and Lentini et al. (2012) have demonstrated that *Clostridium-*enriched cultures are capable of extensive reduction of goethite- and hematite-Fe(III).

### Microbially Induced Hydrologic Alterations of Canga Columns

**FIGURE 5:**
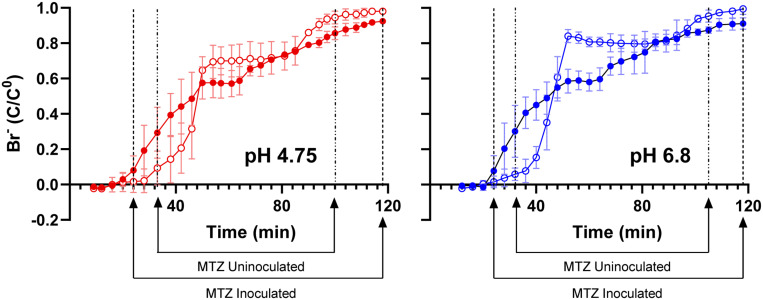
Bromide breakthrough curves of *sub muros*-inoculated (closed shapes) and uninoculated (open shapes) columns after 63 days of operation. The columns that received the basal SPW pH 4.75 media are in red, with the SPW pH 6.8 in blue. Mass transfer zone (MTZ) lines represent initial breakthrough point where bromide-amended SPW is mixing with bromide-free SPW and adsorption exhaustion point where column is saturated with bromide-amended SPW. SPW was fed to columns at a rate of 0.2 mL/min. Error bars represent one standard deviation of triplicate columns.

Based on dissolved Fe(II) in column effluents, approximately 40 mg of Fe(OH)_3_ were removed from the columns due to microbiological Fe(III) reduction (Table 1). To determine if this export of mass impacted the hydraulic properties of the columns, we pumped bromide-amended SPW through the columns. Bromide breakthrough in the *sub muros*-inoculated columns preceded that of the uninoculated columns, and breakthrough was spread out in comparison to that of the uninoculated columns, which had a sharper curve (Figure 5). These observations indicate that flow through the uninoculated columns did not experience the same mass transfer resistance seen in the columns in which microbiological Fe(III) reduction occurred (Lassabatere et al., 2004; Koestel et al., 2011; Safadoust et al., 2016). The porosity that allowed earlier bromide breakthrough is due to reductive dissolution of Fe(III) phases and export of dissolved Fe(II). In similar column experiments, Liang et al. (2019) found that bioreduction of sediment-associated Fe(III) led to the structural breakdown of particles in the columns and led to the earlier breakthrough of poorly-diffusible 2,6-difluorobenzoate. No change in more diffusible bromide breakthrough was observed after Fe(III) bioreduction (Liang et al., 2019). In the work presented here, Fe(III) bioreduction was more extensive, with maximal effluent Fe(II) concentrations of approximately 3 mM, in comparison to the maximal Fe(II) concentration of 0.3 mM observed by Liang et al. (2019). Taken together, the extensive Fe(III) bioreduction observed in these column experiments induced changes to the water flow paths in the packed canga.

### Biogeochemical Implications

The results of our experiments indicate that the Fe(III) reducing activities of microorganisms associated with IFCs can induce reductive dissolution of Fe(III) phases, resulting in the transport of dissolved Fe(II) and hydrologic changes that are consistent with cave formation. While the Fe(III)-rich rocks of this region were generally considered to be resistant to weathering (Schuster et al., 2012; Monteiro et al., 2014), it is becoming increasingly clear that microbiological activities may induce extensive transformations to these rocks, especially canga (Parker et al., 2013, 2018; Levett et al., 2016, 2020; Gagen et al., 2018, 2019; Paz et al., 2020). Previous work has focused on the transformations of canga-Fe as a mechanism of canga permanence, whereby the weathering resistance of canga is owed to the alternating reductive dissolution of Fe(III) (hydr)oxides and abiotic or microbiological reoxidation of Fe(II) back to Fe(III) (Levett et al., 2016, 2020; Gagen et al., 2018, 2019, 2020; Paz et al., 2020). In this way, canga appears to be continuously weathering and reforming. The work here indicates that the Fe(III) rich phases could be more extensively weathered and removed from the systems, driven by the increased rates of Fe-reduction induced by water flow. Thus, Fe may be extensively mobilized from rocks in the SE and QF by microbiological weathering via microbial Fe(III) reduction (either through respiratory activity or as an electron sink) and separation, which can be enhanced by groundwater flow. These results should be applicable to other iron formation areas in Brazil and help explain why caves are larger in the iron deposits of Carajás, in the wetter Amazon Basin (Auler et al., 2019). A positive feedback mechanism, in which fast infiltration water would lead to increased porosity and thus even faster water percolation could operate, enhancing the mass transfer mechanisms required to mobilize Fe(II). Our observations indicate that microorganisms associated with these systems are capable of robust Fe(III) reducing activity, which could induce sufficient reductive dissolution of Fe(III) phases to form a cave. The numerous caves of the SE and QF (>3,000; Auler et al., 2019) indicate that the activity is extensive and continuously occurring. Indeed, we have observed remarkably high dissolved Fe concentrations in water circulating around caves in the QF (Parker et al., 2018). This extensive weathering of SE and QF Fe(III) phases may represent a previously underappreciated component of regional, and perhaps global Fe budgets.

## Data Availability Statement

The datasets presented in this study can be found in online repositories. The names of the repository/repositories and accession number(s) can be found in the article/supplementary material.

## Author Contributions

KC, MM, and TR carried out the lab work. JS, AA, CP, and HB carried out the fieldwork. All authors were involved in the design of the experiments and contributed to the manuscript.

## Conflict of Interest

The authors declare that the research was conducted in the absence of any commercial or financial relationships that could be construed as a potential conflict of interest.
